# Expert consensus on the off-label use in China of drugs for rare hematologic diseases (2024 edition)

**DOI:** 10.3389/fphar.2024.1477550

**Published:** 2024-11-22

**Authors:** Boxin Zhao, Xuan Zhou, Ping Zheng, Bo Zhang, Xiaoqin Feng, Jie Chen, Lisheng Cai, Yilu Chen, Liya He, Jianfen Su, Shuqin Cheng, Yingtong Zeng, Guowei Li, Bo Ji, Jianlong Wu, Weiyi Feng, Maobai Liu, Yiran Jin, Taotao Liu, Xiaolan Mo, Junyan Wu, Hui Wu, Hongliang Zhang, Zhichang Zheng, Zhihua Zheng, Jing Sun, Yilei Li, Guangdong Pharmaceutical Association, Hematology Group of Rare Disease Expert Committee of Guangdong Pharmaceutical Association

**Affiliations:** ^1^ Department of Pharmacy, Nanfang Hospital, Southern Medical University, Guangzhou, China; ^2^ Clinical Pharmacy Center, Nanfang Hospital, Southern Medical University, Guangzhou, China; ^3^ Department of Hematology, Nanfang Hospital, Southern Medical University, Guangzhou, China; ^4^ Department of Pharmacy, Peking Union Medical College Hospital, Beijing, China; ^5^ State Key Laboratory of Complex Severe and Rare Diseases, Peking Union Medical College Hospital, Beijing, China; ^6^ Department of Pediatrics, Nanfang Hospital, Southern Medical University, Guangzhou, China; ^7^ Department of Pharmacy, The First Affiliated Hospital of Sun Yat-sen University, Guangzhou, China; ^8^ Department of Hematology, Shenzhen Second People’s Hospital, Shenzhen, China; ^9^ Department of Pharmacy, Guangzhou Women and Children’s Medical Center, Guangzhou, China; ^10^ Department of Pediatrics, Guangzhou Women and Children’s Medical Center, Guangzhou, China; ^11^ Department of Pharmacy, Panyu Central Hospital, Guangzhou, China; ^12^ Department of Hematology, Panyu Central Hospital, Guangzhou, China; ^13^ Department of Pharmacy, Guangdong Provincial People’s Hospital, Southern Medical University, Guangzhou, China; ^14^ Department of Hematology, Huizhou Central People’s Hospital, Huizhou, China; ^15^ Department of Clinical Pharmacy, General Hospital of Southern Theatre Command of PLA, Guangzhou, China; ^16^ Department of Pharmacy, Shenzhen Second People’s Hospital, Shenzhen, China; ^17^ Department of Pharmacy, The First Affiliated Hospital of Xi’an Jiaotong University, Xi’an, Shaanxi, China; ^18^ Department of Pharmacy, Fujian Medical University Union Hospital, Fuzhou, China; ^19^ Department of Pharmacy, The Second Hospital of Hebei Medical University, Shijiazhuang, China; ^20^ Pharmacy Department, The First Affiliated Hospital of Guangxi Medical University, Nanning, China; ^21^ Department of Pharmacy, Sun Yat-Sen Memorial Hospital, Sun Yat-Sen University, Guangzhou, China; ^22^ Department of Pharmacy, First Affiliated Hospital of Kunming Medical University, Kunming, China; ^23^ Department of Pharmacy, The Affiliated Hospital of Guizhou Medical University, Guiyang, China; ^24^ Guangdong Pharmaceutical Association, Guangzhou, China

**Keywords:** rare diseases, hematological disease, off-label drug use, expert consensus, evidence based pharmacy

## Abstract

Drug package inserts are a crucial foundation for clinical medication practices and serve as the legal basis for guiding rational drug use and ensuring patient safety and efficacy. As rare disease treatments evolve, current package inserts often need to meet the clinical requirements for treating such conditions, frequently resulting in off-label drug use. This consensus is derived from discussions between Guangdong Pharmaceutical Association Hematologic Rare Diseases Group experts. The consensus aims to provide a framework and reference for the clinical application of off-label drug use in treating rare hematologic diseases.

## 1 Introduction

Package inserts are crucial for the use of clinical medications and serve as the legal standard that guides clinicians and pharmacists toward rational drug use. Despite rapid advances in diagnosing and treating rare diseases, updates to package inserts lag, leading to widespread off-label drug use in clinical settings. This is often unavoidable due to the unique characteristics of the rare disease patient population. Article 29, Clause 2 of the Chinese “Law on Doctors” states “in the absence of effective or superior treatment methods and under special circumstances, physicians may use drugs not explicitly indicated in the drug instructions but supported by evidence-based medical evidence, with the patient’s informed consent.” (Physician Law on Doctors of the People’s Republic of China, 2021) However, standardized guidelines or expert consensus on off-label use for rare disease patients remain absent. Although the current progress in rare diseases has seen significant advancements, particularly in the areas of gene therapy, immunotherapy, antibody-drug conjugate agents and innovative drugs for rare hematological diseases, there are still obstacles in the accessibility of these drugs or treatments in China. The new use of old drugs still dominates the treatment of rare hematological diseases, however, off-label drug use is common because no updated of the instructions and less clinical trials for rare diseases. To address this, the Guangdong Pharmaceutical Association Rare Disease Expert Committee of the has compiled the “Expert Consensus on Off-Label Use of Drugs for Rare Hematological Diseases (2024 Edition)” (hereafter referred to as the “Consensus”). This document seeks to provide evidence-based guidance for the off-label use of commonly used drugs in the diagnosis and treatment of rare hematologic diseases, standardize related drug use, and improve pharmaceutical supervision and management in the individualized treatment of special populations. The “Consensus” is intended for use by physicians when prescribing and pharmacists when reviewing prescriptions at medical institutions. However, the management of off-label drug use in clinical practice should still adhere to the relevant regulations. Clinicians are urged to assess the benefits and risks of off-label drug use for patients and to avoid such practices whenever satisfactory clinical efficacy can be achieved with standard drug instructions.

## 2 Materials and methods

### 2.1 The consensus scope, target professionals, and target patient population

This consensus applies to healthcare institutions at all levels for treating rare hematologic diseases. The target patient population includes individuals with rare hematologic diseases listed in the first Chinese list of rare diseases. The target healthcare professionals include physicians, pharmacists, nurses, other healthcare workers, and policymakers involved in managing rare diseases.

### 2.2 The methodology of the consensus development

A nominal group technique was used to discuss a specific topic (off-label drug use in treating rare hematologic diseases) in an online conference format organized by an experienced facilitator and attended by 26 relevant experts. The consensus development process and reporting adhered to the World Health Organization Handbook for Guideline Development (2^nd^ edition) (Word Health Organization, 2014), the Statement of Reporting Items for Practice Guidelines in Healthcare (RIGHT) (Chen Y et al., 2017), and the specification of evidence-based pharmaceutical evaluation methods for off-label drug use (T/GDPA 1-2021, 2021, Guangdong Pharmaceutical Association) (The specification of evidence-base pharmaceutical evaluation method for off-label drug use, 2021). Conflicts of interest and disclosures were managed according to the Recommendations for the Conduct, Reporting, Editing, and Publication of Scholarly Work in Medical Journals of the International Committee of Medical Journal Editors.

### 2.3 Consensus panels

The group members comprised experts from the following disciplines: hematologic medicine, clinical pharmacy, and pharmaceutical administration. The composition and positions of the panel members are shown in Supplementary Table S1.

### 2.4 Evidence retrieval and data extraction

The “Consensus” includes drugs commonly used off-label in treating rare hematologic diseases, presented in a tabular format for clarity and organized by therapeutic properties and application fields. The inclusion criteria for these drugs are based on the “2023 Guangdong Pharmaceutical Association Off-Label Drug Use Directory” (Guangdong Pharmaceutical Association, 2023), with adjustments for the unique aspects of rare disease medications: (1) included in package inserts from the United States, Europe, or Japan; (2) listed in the “Chinese Pharmacopoeia Clinical Medication Instructions” or “Clinical Diagnosis and Treatment Guidelines” (published by the Chinese Medical Association and People’s Medical Publishing House); (3) included in leading international and Chinese guidelines or consensus documents; (4) rated by Micromedex^®^ with an efficacy rating and recommendation level IIb or evidence level C or above; and (5) supported by published randomized controlled trial studies in first-quartile (Q1) SCI journals of the relevant field.

When Micromedex^®^ lacks evaluations for certain off-label drugs commonly used in rare diseases, the consensus adopts the Thomson Micromedex^®^ classification system standards to assess the efficacy, recommendation, and evidence levels of the included drugs, as described by the expert drafting group. Details of this classification system are provided in Supplementary Table S1.

### 2.5 Comprehensive analysis of evidence and compilation of evidence report

After analyzing and summarizing the retrieved data, expert opinions were gathered through meetings and online consultations to supplement the evidence. Finally, the writing team prepared an evidence report, which was reviewed by the consensus conference.

### 2.6 The process of formulating recommendations

The writing team developed recommendations based on the best available evidence. When direct supportive evidence was insufficient, expert clinical experience was collected online to supplement recommendations. After discussions of expert evidence, the team formulated an initial draft of the expert consensus. The guideline members provided feedback on recommendations and the expert consensus statement, ultimately establishing linguistic consensus.

The “Consensus” is structured in a tabular format to enhance clarity and conciseness. Each entry in the consensus table includes the following elements: “Generic Name,” specifying the drug’s official generic designation; “Dosage Form,” detailing the dosage form such as tablet or injection form; “Off-Label Type,” describing how the drug is used beyond its approved indications; “Off-Label Content,” providing specific details on the conditions or symptoms treated off-label; “Specific Usage,” outlining the dosage and administration details for off-label use; “Evidence and References,” including citations that support off-label use; and “Evidence Level,” indicating the grade or level of evidence supporting the drug’s efficacy and safety for off-label use.

## 3 Result

### 3.1 Off-label drug use for hemophilia treatment

Hemophilia, a rare X-linked recessive hereditary bleeding disorder, is categorized into Hemophilia A and Hemophilia B. Hemophilia A results from a deficiency of clotting factor VIII. At the same time, Hemophilia B is due to a deficiency of clotting factor IX, each due to mutations in their respective genes. The prevalence of hemophilia in China is approximately 2.73–3.09 per 100,000 (Xue and Yang, 2022). Primary treatment approaches include plasma-derived or recombinant clotting factor replacement, non-factor replacement, and gene therapy. However, there are no approved indications in the drug package inserts for immune tolerance therapy for Factor VIII inhibitors or the hemostatic treatment of low-titer Factor VIII inhibitors (Xue F, et al., 2023). Supplementary Table S2 presents the expert consensus on off-label drug use in the treatment of hemophilia.

### 3.2 Off-label drug use for Castleman’s disease treatment

Castleman’s disease (CD), or giant lymph node hyperplasia or angiofollicular lymph node hyperplasia, is a relatively rare lymphoproliferative disorder. The generally accepted pathogenic mechanisms of CD mainly involve cytokine interleukin-6 (IL-6), human herpesvirus 8 (HHV-8), and human immunodeficiency virus (HIV) infection. The incidence rate is approximately 0.2 per 10,000, although no data is available on the incidence of CD in China (Zhang L, et al. 2023). According to current guidelines and consensus, targeting IL-6 is the preferred first-line treatment for newly diagnosed idiopathic multicentric Castleman’s disease (iMCD). However, IL-6 targeted therapy is not universally effective, achieving an efficacy rate of only 34% in randomized controlled trials (RCTs). Siltuximab, the only IL-6-targeted drug approved in China for iMCD, is costly and increases the economic burden on patients. Additionally, drug-targeting pathways beyond IL-6 have yet to receive approval in China. Table 1 presents the expert consensus on off-label drug use to treat Castleman’s disease.

**TABLE 1:** Off-label drug usage catalog for Castleman’s disease treatment.

Generic name	Dosage form	Off-label type	Off-label content	Specific usage	Evidence and references	Evidence level
Tocilizumab	Injection	Indication	Castleman’s disease (UCD and iMCD)	In countries where siltuximab is not available or approved, tocilizumab can be used	International evidence-based consensus treatment guidelines for idiopathic multicentric Castleman disease (van Rhee F, et al., 2018) International consensus diagnostic and treatment guidelines for unicentric Castleman disease (van Rhee F, et al., 2020)	Not listed in Micromedex
Rituximab	Injection	Indication	Castleman’s disease (UCD compression symptoms that cannot be completely resected and severe or nonsevere iMCD)	For patients unresponsive to IL-6 monoclonal antibody therapy, consider a regimen based on rituximab plus corticosteroids ± immunomodulators/immunosuppressants (second- or third-line treatments include thalidomide, cyclosporin A, sirolimus, or bortezomib)	International evidence-based consensus treatment guidelines for idiopathic multicentric Castleman disease (van Rhee F, et al., 2018) International consensus diagnostic and treatment guidelines for unicentric Castleman disease (van Rhee F, et al., 2020)	Not listed in Micromedex
Prednisone	Tablet	Indication	Castleman’s disease (UCD compression symptoms that cannot be completely resected and severe or non severe iMCD)	Same as rituximab	International evidence-based consensus treatment guidelines for idiopathic multicentric Castleman disease (van Rhee F, et al., 2018) International consensus diagnostic and treatment guidelines for unicentric Castleman disease (van Rhee F, et al., 2020)	Not listed in Micromedex
Thalidomide	Tablet	Indication	Castleman’s disease (UCD compression symptoms that cannot be completely resected and severe or non severe iMCD)	Same as rituximab	International evidence-based consensus treatment guidelines for idiopathic multicentric Castleman disease (van Rhee F, et al., 2018) International consensus diagnostic and treatment guidelines for unicentric Castleman disease (van Rhee F, et al., 2020) Chinese Consensus on Diagnosis and Treatment of Castleman’s Disease (2021) (Hematology Committee of Chinese Medical Association, et al., 2021)	Not listed in Micromedex
Cyclosporine	Injection/Capsule/Oral Solution	Indication	Castleman’s disease (Combination therapy for iMCD-TAFRO)	Same as rituximab	International evidence-based consensus treatment guidelines for idiopathic multicentric Castleman disease (van Rhee F, et al., 2018) International consensus diagnostic and treatment guidelines for unicentric Castleman disease (van Rhee F, et al., 2020) Chinese Consensus on Diagnosis and Treatment of Castleman’s Disease (2021) (Hematology Committee of Chinese Medical Association, et al., 2021)	Not listed in Micromedex
Sirolimus	Tablet/Capsule/Oral Solution	Indication	Castleman’s disease (Second line treatment of non severe iMCD)	Same as rituximab	International evidence-based consensus treatment guidelines for idiopathic multicentric Castleman disease (van Rhee F, et al., 2018) International consensus diagnostic and treatment guidelines for unicentric Castleman disease (van Rhee F, et al., 2020) Chinese Consensus on Diagnosis and Treatment of Castleman’s Disease (2021) (Hematology Committee of Chinese Medical Association, et al., 2021)	Not listed in Micromedex
Bortezomib	Injection	Indication	Castleman’s disease (Second line treatment of non severe iMCD)	Same as rituximab	International evidence-based consensus treatment guidelines for idiopathic multicentric Castleman disease (van Rhee F, et al., 2018) International consensus diagnostic and treatment guidelines for unicentric Castleman disease (van Rhee F, et al., 2020) Chinese Consensus on Diagnosis and Treatment of Castleman’s Disease (2021) (Hematology Committee of Chinese Medical Association, et al., 2021)	Not listed in Micromedex

### 3.3 Off-label drug use for Erdheim-Chester disease treatment

Erdheim-Chester disease (ECD) is a rare form of non-Langerhans cell histiocytosis known as lipid granulomatosis. This disease predominantly affects middle-aged and older individuals, with no marked gender differences in incidence rates. ECD can involve the skeletal system and multiple organs, most frequently affecting the diaphyseal and metaphyseal regions of the long bones, particularly in the lower extremities. Approximately 1,000 cases of ECD have been reported globally, yet comprehensive epidemiological data is lacking in China (Merai H, et al., 2020). There are no RCTs investigating treatments for ECD. Glucocorticoids and immunosuppressants have shown effectiveness in some cases. Additionally, BRAF inhibitors are applicable to patients with BRAF mutations, and other kinase inhibitors have also demonstrated efficacy in patients without such mutations. Interferon-alpha is commonly used to treat this condition, and recent studies highlight the potential of TNF-alpha antagonists, IL-6 antagonists, and IL-1 antagonists (Haroche J, et al., 2020). However, these therapeutic agents have not received approval for these specific indications. Table 2 presents the expert consensus on the off-label use of these drugs to treat ECD.

**TABLE 2:** Off-label drug usage catalog for erdheim-chester disease treatment.

Generic name	Dosage form	Off-label type	Off-label content	Specific usage	Evidence and references	Evidence level
Vemurafenib	Tablet	Indication	Erdheim-Chester disease	Treatment for Erdheim-Chester disease with BRAF V600 mutation, 480–960 mg orally, twice a day	FDA approved (Product Information: ZELBORAFR oral tablets, vemurafenib oral tablets, 2017) Consensus guidelines for the diagnosis and clinical management of Erdheim-Chester disease (Diamond EL, et al., 2014)	Not listed in Micromedex
Interferon-α	Injection	Indication	Erdheim-Chester disease	3 mIU subcutaneous injection, three times a week (standard dose); or 6–9 mIU subcutaneous injection, 3 days a week (high dose)	Consensus guidelines for the diagnosis and clinical management of Erdheim-Chester disease (Diamond EL, et al., 2014)	Not listed in Micromedex
Pegylated Interferon-α	Injection	Indication	Erdheim-Chester disease	Subcutaneous injection of 135 μg/week (standard dose), or 180 μg/week (high dose)	Consensus guidelines for the diagnosis and clinical management of Erdheim-Chester disease (Diamond EL, et al., 2014)	Not listed in Micromedex
Trametinib	Tablet	Indication	Erdheim-Chester disease	Used for the combined treatment of Erdheim-Chester disease with BRAF V600 and KRAS Q61H mutations	Trametinib after disease reactivation under dabrafenib in Erdheim-Chester disease with both BRAF and KRAS mutations (Nordmann et al., 2017)	Not listed in Micromedex
Cladribine	Injection	Indication	Erdheim-Chester disease	6 mg/m2 intravenous injection, once a day for 5 consecutive days, every 4 weeks	Consensus guidelines for the diagnosis and clinical management of Erdheim-Chester disease (Diamond EL, et al., 2014)	Not listed in Micromedex
Sirolimus	Tablet/Capsule/Oral Solution	Indication	Erdheim-Chester disease	2 mg/day	Sirolimus plus prednisone for Erdheim-Chester disease: an open-label trial (Gianfreda et al., 2015) Consensus guidelines for the diagnosis and clinical management of Erdheim-Chester disease (Diamond EL, et al., 2014)	Not listed in Micromedex
Prednisone	Tablet	Indication	Erdheim-Chester disease	Initial dose of 0.75 mg/kg/day, gradually reducing to 0.125–0.5 mg/kg/day over 6 months	Sirolimus plus prednisone for Erdheim-Chester disease: an open-label trial (Gianfreda et al., 2015)	Not listed in Micromedex

### 3.4 Off-label drug use for fanconi anemia treatment

Fanconi anemia is a rare genetic blood disorder that predominantly manifests in childhood, with an incidence rate of approximately 1 in 136,000 (Che R, et al., 2018). Androgens have been found to improve blood cell counts in approximately 50% of affected patients. However, androgen treatments currently available in China lack approval for this specific therapeutic indication. Table 3 presents the expert consensus on the off-label use of androgen medications for Fanconi anemia.

**TABLE 3:** Off-label drug usage catalog for fanconi anemia treatment.

Generic name	Dosage form	Off-label type	Off-label content	Specific usage	Evidence and references	Evidence level
Danazol	Capsule	Indication	Fanconi Anemia	Initial dosage of 200 mg per dose, 2–3 times per day. Once effective, the maintenance dosage is generally 50% or less of the initial dosage, decreased over intervals of 1–3 months or longer	Androgens and liver tumors: Fanconi’s anemia and non-Fanconi’s conditions (Velazquez and Alter, 2004)	Not listed in Micromedex

### 3.5 Off-label drug use for langerhans cell histiocytosis treatment

Langerhans cell histiocytosis (LCH) is a group of histiocyte proliferative disorders characterized by an unknown etiology. It is traditionally categorized into three clinical types: Letterer-Siwe disease (L-S disease), Hand-Schüller-Christian disease (H-S-C disease), and eosinophilic granuloma (EGB), each marked by the pathological proliferation of Langerhans cells. The incidence rate among children is approximately 3–5 per million, whereas in adults, it is less than 1 to 2 cases per million (Baumgartner I, et al., 1997). There are no widely accepted treatment recommendations for adult patients. Recent advances in chemotherapy have significantly improved the prognosis of this disease. However, these chemotherapeutic agents have not received approval for the indication of LCH in China (Dai and Cao, 2023). Table 4 presents the expert consensus on the off-label use of drugs for LCH.

**TABLE 4:** Off-label drug usage catalog for langerhans cell histiocytosis treatment.

Generic name	Dosage form	Off-label type	Off-label content	Specific usage	Evidence and references	Evidence level
Vincristine	Injection	Indication	Langerhans Cell Histiocytosis	Initial dosage of 3.7 mg/m^2^ via intravenous injection. Subsequent doses are administered with increasing quantities: the second dose is 5.5 mg/m^2^, the third is 7.4 mg/m^2^, the fourth is 9.25 mg/m^2^, and the fifth is 11.1 mg/m^2^, weekly to maximum of 18.5 mg/m^2^	FDA approved (Product Information: vinblastine sulfate intravenous injection, 2008)	Effectiveness Class IIa Recommendation Class IIa Evidence Level Category B
Cladribine	Injection	Indication	Langerhans Cell Histiocytosis (Pediatric)	In a study involving ten children with recurrent or refractory Langerhans cell histiocytosis, the initial dose of cladribine was 5 mg/m^2^ per day, administered for 3 days, then increased to 6.5 mg/m^2^ for another 3 days per cycle, for up to six cycles. The cladribine was mixed with 100 mL of saline and administered via a portable infusion pump Cladribine was effectively used to treat six children with recurrent Langerhans cell histiocytosis. The treatment involved administering cladribine intravenously at doses of 5–7 mg/m^2^ per day for five consecutive days, with cycles repeating every 21–28 days. The patients underwent six treatment cycles	Efficacy studies on pediatric patients with refractory Langerhans cell histiocytosis (Stine KC, et al., 2004) Treatment of children with Langerhans cell histiocytosis with 2-chlorodeoxyadenosine (Rodriguez-Galindo C, et al., 2002) Analysis of outcome for patients with mass lesions of the central nervous system due to Langerhans cell histiocytosis treated with 2-chlorodeoxyadenosine (Dhall G, et al., 2008)	Effectiveness Class IIa Recommendation Class IIb Evidence Level Category B
Mechlorethamine	Tincture/Ointment	Indication	Langerhans Cell Histiocytosis	Diluted in water to 200 mg/L (0.02%). Applied topically with a swab, initial dose 2–3 mg/day, wash off after 10 min. Apply daily until lesions recede, then reduce frequency to every 2 days, then every 3 days, and weekly until clear	Long term follow up of topical Mechlorethamine treatment for cutaneous Langerhans cell histiocytosis (Hoeger PH, et al., 2000)	For adults: effectiveness Class IIa, recommendation Class IIb, evidence category C For children: effectiveness Class IIa, recommendation Class IIb, and evidence category B
Cyclophosphamide	Injection/Tablet	Indication	Langerhans Cell Histiocytosis	300 mg/m^2^ on days 1, 8, and 15 of a 4-week cycle	Phase 2 study of oral thalidomide-cyclophosphamide-dexamethasone for recurrent/refractory adult Langerhans cell histiocytosis (Wang JN, et al., 2022)	Effectiveness Class IIa, Recommendation Class IIb, Evidence Level Category B
Etanercept	Injection	Indication	Langerhans Cell Histiocytosis	0.4 mg/kg twice weekly	Successful treatment study of Langerhans'-cell histiocytosis with etanercept (Henter JI, et al., 2001)	Effectiveness Class IIa, Recommendation Class IIb, Evidence Level Category C
Cyclosporine	Injection/Capsule/Oral Solution	Indication	Langerhans Cell Histiocytosis	12 mg/kg/day; 15–20 mg/kg/day	Multisystem Langerhans-cell histiocytosis with life-threatening pulmonary involvement--good response to cyclosporine A (Zeller B, et al., 2000) Treatment of relapsed Langerhans cell histiocytosis by cyclosporin A combined with etoposide and prednisone (Korholz D, et al., 1997) Cyclosporine therapy for advanced Langerhans cell histiocytosis (Mahmoud HH, et al., 1991)	Effectiveness Class IIa, Recommendation Class IIb, Evidence Level Category C
Etoposide	Injection/Capsule	Indication	Langerhans Cell Histiocytosis	100 mg/m^2^ twice weekly for 4 weeks, then once every 2–4 weeks	Etoposide in the treatment of six children with Langerhans cell histiocytosis (histiocytosis X) (Viana MB, et al., 1991) Treatment of Langerhans cell histiocytosis in children with etoposide (Ishii et al., 1992)	Effectiveness Class IIa, Recommendation Class IIb, Evidence Level Category B
Pamidronate Disodium	Injection	Indication	Pain management in Langerhans Cell Histiocytosis	90 mg IV per session, followed by monthly sessions for 4 months	Pamidronate for bone pain from osteolytic lesions in Langerhans'-cell histiocytosis (Arzoo K, et al., 2001) 2. Treatment of Langerhans cell histiocytosis with pamidronate (Farran RP, et al., 2001)	Effectiveness Class IIa, Recommendation Class IIb, Evidence Level Category C

### 3.6 Off-label drug use for paroxysmal nocturnal hemoglobinuria treatment

Paroxysmal nocturnal hemoglobinuria (PNH) is a disorder resulting from acquired mutations in the PIG-A gene of hematopoietic stem cells, which leads to increased sensitivity of blood cells to complement. This increased sensitivity causes intravascular hemolysis, thrombosis, and bone marrow failure. The annual global incidence rate of PNH is approximately 1–10 per million (Brodsky RA, 2014). The primary treatment for symptomatic management currently includes complement C5 inhibitors, such as eculizumab. However, there is still off-label use of other medications for the symptomatic treatment of PNH. Table 5 shows the expert consensus on the off-label drug use for PNH.

**TABLE 5:** Off-label drug usage catalog for paroxysmal nocturnal hemoglobinuria treatment.

Generic name	Dosage form	Off-label type	Off-label content	Specific usage	Evidence and references	Evidence level
Deferiprone	Tablet/Oral Solution	Indication	Paroxysmal Nocturnal Hemoglobinuria	3–6 g/day	Long-term treatment of transfusional iron overload with the oral iron chelator deferiprone (L1): a Dutch multi-center trial (Kersten MJ, et al., 1996)	Not listed in Micromedex

### 3.7 Off-label drug use for POEMS syndrome

POEMS syndrome is a multisystem disorder associated with plasma cell disease, characterized clinically by polyneuropathy, organomegaly, endocrinopathy, monoclonal proteinemia, and skin changes. The prevalence rate is estimated to be approximately 0.3 per 100,000 (Soubrier MJ, et al., 1994; Li J, et al., 2011; Kulkarni et al., 2011), and as a group of clinical disorders attributable to plasma cell malignancies. To date, no RCTs have been reported for the treatment of POEMS syndrome, with treatment recommendations derived primarily from case reports. Table 6 presents the expert consensus on the off-label use of drugs to treat POEMS syndrome.

**TABLE 6:** Off-label drug usage catalog for POEMS syndrome.

Generic name	Dosage form	Off-label type	Off-label content	Specific usage	Evidence and references	Evidence level
Lenalidomide	Capsule	Indication	POEMS Syndrome	High-dose treatment: 25 mg/day for 3 weeks, 1 week off, for 6 cycles with dexamethasone; Low-dose treatment: 10 mg/day with dexamethasone	1. Lenalidomide and dexamethasone in patients with POEMS syndrome: results of a prospective open-label trial. (Nozza A, et al., 2017) 2. Efficacy and safety of low-dose lenalidomide plus dexamethasone in patients with relapsed or refractory POEMS syndrome. (Cai QQ, et al., 2015)	Effectiveness Class IIa, Recommendation Class IIb, Evidence Category B
Thalidomide	Tablet	Indication	POEMS Syndrome	Combined with dexamethasone, a 28-day cycle, where dexamethasone is used at 12 mg/m^2^/day for the first 4 days of the cycle; Thalidomide starts at 100 mg every other day, increasing to 100 mg/day from day 8, and 200 mg/day from day 15	1. European myeloma network recommendations on diagnosis and management of patients with rare plasma cell dyscrasias. (Gavriatopoulou M, et al., 2018) 2. POEMS syndrome: 2021 Update on diagnosis risk-stratification and management.(Dispenzieri A, 2021) 3. Safety and efficacy of thalidomide in patients with POEMS syndrome: a multicentre randomised double-blind placebo-controlled trial.(Diamond et al., 2014)	Not listed in Micromedex
Mephalan	Injection/Tablet	Indication	POEMS Syndrome	Combined with dexamethasone, dosage of 140–200 mg/m^2^, lower doses used for more severe cases	1. European myeloma network recommendations on diagnosis and management of patients with rare plasma cell dyscrasias. (Gavriatopoulou M, et al., 2018) 2. POEMS syndrome: 2021 Update on diagnosis risk-stratification and management. (Dispenzieri A, 2021)	Not listed in Micromedex
Bortezomib	Injection	Indication	POEMS Syndrome	Combined with cyclophosphamide and dexamethasone: Bortezomib administered on days 1, 4, and 5 at 1 mg/m^2^ IV; Cyclophosphamide 200 mg on days 8 and 11; Dexamethasone 20 mg from days 1–4 and 8 to 11	1. European myeloma network recommendations on diagnosis and management of patients with rare plasma cell dyscrasias. (Gavriatopoulou M, et al., 2018) 2. POEMS syndrome: 2021 Update on diagnosis risk-stratification and management. (Dispenzieri, 2021) 3. Successful treatment of newly diagnosed POEMS syndrome with reduced-dose bortezomib based regimen.(Nordmann et al., 2017)	Not listed in Micromedex
Lenalidomide	Capsule	Indication	POEMS Syndrome	High-dose treatment: 25 mg/day for 3 weeks, then pause for 1 week, for 6 cycles in combination with dexamethasone; Low-dose treatment: 10 mg/day in combination with dexamethasone	1. Lenalidomide and dexamethasone in patients with POEMS syndrome: results of a prospective open-label trial. (Nozza et al., 2017) 2. Efficacy and safety of low-dose lenalidomide plus dexamethasone in patients with relapsed or refractory POEMS syndrome. (Cai et al., 2015)	Effectiveness Class IIa, Recommendation Class IIb, Evidence Category B
Thalidomide	Tablet	Indication	POEMS Syndrome	Combined with dexamethasone, a 28-day cycle, where dexamethasone is used at 12 mg/m^2^/day for the first 4 days of the cycle; Thalidomide starts at 100 mg every other day, increasing to 100 mg/day from day 8, and 200 mg/day from day 15	1. European myeloma network recommendations on diagnosis and management of patients with rare plasma cell dyscrasias. (Gavriatopoulou et al., 2018) 2. POEMS syndrome: 2021 Update on diagnosis risk-stratification and management. (Dispenzieri, 2021) 3. Safety and efficacy of thalidomide in patients with POEMS syndrome: a multicentre randomised double-blind placebo-controlled trial.(Misawa et al., 2016)	Not listed in Micromedex
Mephalan	Injection/Tablet	Indication	POEMS Syndrome	Combined with dexamethasone, dosage of 140–200 mg/m^2^, lower doses used for more severe cases	1. European myeloma network recommendations on diagnosis and management of patients with rare plasma cell dyscrasias. (Gavriatopoulou et al., 2018) 2. POEMS syndrome: 2021 Update on diagnosis risk-stratification and management. (Dispenzieri, 2021)	Not listed in Micromedex
Bortezomib	Injection	Indication	POEMS Syndrome	Combined with cyclophosphamide and dexamethasone: Bortezomib administered on days 1, 4, 8, and 11 at 1 mg/m^2^ IV; Cyclophosphamide 200 mg on days 8 and 11; Dexamethasone 20 mg from days 1–4 and 8 to 11	1. European myeloma network recommendations on diagnosis and management of patients with rare plasma cell dyscrasias. (Gavriatopoulou et al., 2018) 2. POEMS syndrome: 2021 Update on diagnosis risk-stratification and management. (Dispenzieri A, 2021) 3. Successful treatment of newly diagnosed POEMS syndrome with reduced-dose bortezomib based regimen.(He et al., 2018)	Not listed in Micromedex
Daratumumab	Injection	Indication	POEMS Syndrome	16 mg/kg once a week	POEMS syndrome: 2021 Update on diagnosis risk-stratification and management. (Dispenzieri, 2021)	Not listed in Micromedex
Dexamethasone	Injection/Tablet	Indication	POEMS Syndrome	Used in combination with other chemotherapy drugs	1. Update on the Diagnosis and Treatment of POEMS (Polyneuropathy Organomegaly Endocrinopathy Monoclonal Gammopathy and Skin Changes) Syndrome: A Review (Khouri et al., 2021) 2. POEMS syndrome: 2021 Update on diagnosis risk-stratification and management. (Dispenzieri, 2021) 3. Safety and efficacy of thalidomide in patients with POEMS syndrome: a multicentre randomised double-blind placebo-controlled trial. (Misawa et al., 2016) 4. Successful treatment of newly diagnosed POEMS syndrome with reduced-dose bortezomib based regimen(He et al., 2018)	Not listed in Micromedex

### 3.8 Off-label drug use for treating porphyria

Porphyria includes a group of metabolic disorders resulting from abnormalities in the production and excretion of porphyrins, often influenced by genetic factors. In adults, the most common types are porphyria cutanea tarda, acute intermittent porphyria, and erythropoietic protoporphyria, with incidence rates varying between the different types. There is no epidemiological data available on the prevalence of these disorders in China (Chinese Society of HematologyRed Blood Cell Disease Study Group, 2020). Table 7 presents the expert consensus on the off-label use of drugs to treat porphyria.

**TABLE 7:** Off-label drug usage catalog for treating porphyria.

Generic name	Form	Off-label type	Off-label content	Specific usage	Evidence base and references	Evidence level
Hydroxychloroquine	Tablet	Indication	Alternative treatment for porphyria patients who cannot undergo or tolerate venesection	100 mg per dose, twice a week	Consensus of Chinese Experts on Diagnosis and Treatment of Porphyria (2020), (Chinese Society of Hematology Red Blood Cell Disease Study Group, (2020)	Not listed in Micromedex

### 3.9 Off-label drug use for treating primary light Chain Amyloidosis

Primary light chain amyloidosis is a systemic disease characterized by the deposition of monoclonal immunoglobulin light chains, which possess an antiparallel β-sheet structure in organ tissues, leading to organ dysfunction. The annual incidence rate is estimated to be between 3 and 5 per 1,000,000 people (Kyle RA, et al., 1992), with a higher prevalence observed in males than in females. The clinical manifestations are varied and may include foamy urine, shortness of breath following activity, edema, and discomfort in the liver area. Treatment options include hematopoietic stem cell transplantation, chemotherapy, and supportive care. Chemotherapy regimens commonly use agents such as bortezomib, melphalan, and immunomodulatory drugs (Hematology Oncology Committee of China Anti-Cancer Association, Leukemia & Lymphoma Group Society of Hematology at Chinese Medical Association, 2016). However, these therapeutic drugs have not yet received approval for these specific indications in China. Table 8 presents the expert consensus on the off-label use of drugs for primary light-chain amyloidosis.

**TABLE 8:** Off-label drug usage catalog for treating primary light chain amyloidosis.

Generic name	Form	Off-label type	Off-label content	Specific usage	Evidence base and references	Evidence level
Bortezomib	Injection	Indication	Primary light chain amyloidosis	Used in combination with dexamethasone and/or cyclophosphamide as part of a chemotherapy regimen	1. Management of systemic AL amyloidosis: Recommendations of the Myeloma Foundation of Australia Medical and Scientific (Weber et al., 2015) 2. Guidelines on the management of AL amyloidosis. (Wechalekar et al., 2015) 3. Induction therapy with bortezomib and dexamethasone followed by autologous stem cell transplantation versus autologous stem cell transplantation alone in the treatment of renal AL amyloidosis: A randomized controlled trial. (Huang et al., 2014)	Effectiveness Class IIa, Recommendation Class I Evidence Category B
Thalidomide	Tablet	Indication	Primary light chain amyloidosis	Start with oral thalidomide 200 mg at night. If tolerated, increase dose by 200 mg/day every 2 weeks, up to a maximum of 800 mg	Tolerability and efficacy of thalidomide for the treatment of patients with light chain-associated (AL) amyloidosis. (Seldin et al., 2003)	Effectiveness Class IIb, Recommendation Class IIb, Evidence Category B
Lenalidomide	Capsule	Indication	Primary light chain amyloidosis as part of a treatment regimen	Lenalidomide 15 mg daily, orally from day 1–21 of a 28-day cycle, in combination with dexamethasone and/or cyclophosphamide	1. Salvage therapy with lenalidomide and dexamethasone in patients with advanced AL amyloidosis refractory to melphalan bortezomib and thalidomide. (Palladini et al., 2012) 2. A phase II trial of cyclophosphamide lenalidomide and dexamethasone in previously treated patients with AL amyloidosis. (Palladini et al., 2013)	Effectiveness Class IIa, Recommendation Class IIb, Evidence Category B
Melphalan	Injection/Tablet	Indication	Primary light chain amyloidosis as part of a treatment regimen	200 mg/m^2^ combined with autologous bone marrow transplantation	High-dose melphalan and autologous bone marrow transplantation for systemic AL amyloidosis with cardiac involvement. (Moreau et al., 1996)	Effectiveness Class IIb, Recommendation Class IIb, Evidence Category B
Cyclophosphamide	Injection/Tablet	Indication	Primary light chain amyloidosis as part of a treatment regimen	Oral or intravenous cyclophosphamide 300 mg/m^2^ on days 1, 8, 15, 22 of each cycle, combined with bortezomib, for up to 6 cycles	Daratumumab plus CyBorD for patients with newly diagnosed AL amyloidosis: safety run-in results of ANDROMEDA. (Palladini et al., 2020)	Not listed in Micromedex
Daratumumab	Injection	Indication	Primary light chain amyloidosis	Intravenous administration of 16 mg/kg on days 1, 8, 15, 22 of the first two cycles, then once every week for cycles 3–6, and then once every 4 weeks thereafter	1. A prospective phase 2 trial of daratumumab in patients with previously treated systemic light-chain amyloidosis. (Roussel et al., 2020) 2. Daratumumab for systemic AL amyloidosis: prognostic factors and adverse outcome with nephrotic-range albuminuria. (Kimmich et al., 2020)	Effectiveness Class IIa, Recommendation Class IIa, Evidence Category B
Dexamethasone	Injection/Tablet	Indication	Primary light chain amyloidosis	Oral or intravenous dexamethasone 40 mg weekly (starting dose), for up to 6 cycles	Daratumumab plus CyBorD for patients with newly diagnosed AL amyloidosis: safety run-in results of ANDROMEDA. (Palladini et al., 2020)	Not listed in Micromedex
Pomalidomide	Capsule	Indication	Primary light chain amyloidosis as part of a treatment regimen	Used in combination with dexamethasone, 4 mg daily	A phase 2 trial of pomalidomide and dexamethasone rescue treatment in patients with AL amyloidosis. (Palladini et al., 2017)	Effectiveness Class IIa, Recommendation Class IIb, Evidence Category B

### 3.10 Off-label drug use for treating sickle cell anemia

Sickle cell anemia is a hereditary hemoglobinopathy characterized by substituting valine for glutamic acid at the sixth position of the β-globin chain, leading to the formation of sickle hemoglobin, which replaces normal hemoglobin. Clinically, it is associated with chronic hemolytic anemia, increased susceptibility to infections, and recurrent pain crises that cause chronic local ischemia and subsequent organ and tissue damage (Payne et al., 2020; Rees DC, et al., 2010). In China, the incidence of sickle cell anemia is relatively low, and specific prevalence data are not yet available. Treatment primarily involves blood transfusions and symptomatic drug therapy. The use of hydroxyurea and L-glutamine is currently off-label. Table 9 presents the expert consensus on the off-label use of drugs for sickle cell anemia.

**TABLE 9:** Off-label drug usage catalog for treating sickle cell anemia.

Generic name	Dosage form	Off-label type	Off-label content	Specific usage	Evidence and references	Evidence level
Hydroxyurea	Tablet	Indication	Sickle cell anemia (moderate to severe)	Start with a daily oral dose of 15 mg/kg, increase by 5 mg/kg/day every 12 weeks up to a maximum of 35 mg/kg/day based on blood cell count	FDA approved (Product Information: DROXIAR oral capsules, hydroxyurea oral capsules,, 2015)	Effectiveness Class IIa, Recommendation Class IIa, Evidence Category B
L-glutamine	Capsule	Indication	Sickle cell anemia	For weight below 30 kg, 15 g twice daily; for weight 30–65 kg, 10 g twice daily; for weight above 65 kg, 15 g twice daily	FDA approved (Product Information: ENDARITM oral powder, L-glutamine oral powder., 2017)	Effectiveness Class I, Recommendation Class I, Evidence Category B

### 3.11 Off-label drug use for treating eczema, thrombocytopenia, and immunodeficiency syndrome (Wiskott-Aldrich syndrome)

Wiskott-Aldrich syndrome (WAS) is a rare X-linked recessive genetic disorder characterized by eczema, thrombocytopenia, and immune deficiency, accompanied by an increased risk of autoimmune diseases and malignant tumors. Clinically, WAS is relatively rare, with an estimated annual incidence rate ranging from 1 to 10 in 1,000,000 males and rarer in females (Massaad MJ, et al., 2013; Blundell MP, et al., 2010). Allogeneic hematopoietic stem cell transplantation is the only recognized effective treatment for WAS. Other symptomatic treatments used to manage the condition lack approved indications specifically for WAS. Table 10 presents the expert consensus on the off-label use of drugs for AWS.

**TABLE 10:** Off-label drug usage catalog for treating eczema, thrombocytopenia, and immunodeficiency syndrome.

Generic name	Form	Off-label type	Off-label content	Specific usage	Evidence base and references	Evidence level
Immunoglobulin	Injection	Indication	Eczema-Thrombocytopenia-Immunodeficiency Syndrome	Intravenous infusion, used as a replacement therapy to improve immune function	1. FDA approved (Product Information: CUTAQUIGTM subcutaneous solution, immune globulin subcutaneous human-hipp subcutaneous solution., 2021) 2. The Wiskott-Aldrich syndrome (Hans and Thrasher, 2006)	Effectiveness Class IIa, Recommendation Class IIa, Evidence Category B
Prednisone	Tablet	Indication	Eczema-Thrombocytopenia-Immunodeficiency Syndrome	Used for short-term systemic treatment of eczema	The Wiskott-Aldrich syndrome. (Hans and Thrasher, 2006)	Not listed in Micromedex
Recombinant Thrombopoietin	Injection	Indication	Eczema-Thrombocytopenia-Immunodeficiency Syndrome	Used as an adjunctive treatment for thrombocytopenia	Thrombopoietin receptor agonists in hereditary thrombocytopenias.(Rodeghiero et al., 2018)	Not listed in Micromedex
Eltrombopag	Tablet	Indication	Eczema-Thrombocytopenia-Immunodeficiency Syndrome	Oral daily dose of Eltrombopag (9–75 mg), adjust dose to maintain platelet count >50 × 10^9^/L	1. Effects of eltrombopag on platelet count and platelet activation in Wiskott-Aldrich syndrome/X-linked thrombocytopenia.(Gerrits et al., 2015) 2. Thrombopoietin receptor agonists in hereditary thrombocytopenias (Rodeghiero et al., 2018)	Not listed in Micromedex
Romiplostim	Injection	Indication	Eczema-Thrombocytopenia-Immunodeficiency Syndrome	Used for treatment of Eczema-Thrombocytopenia-Immunodeficiency Syndrome when Eltrombopag is ineffective	Thrombopoietin receptor agonists in hereditary thrombocytopenias (Rodeghiero et al., 2018)	Not listed in Micromedex
Busulfan	Injection/Tablet	Indication	Eczema-Thrombocytopenia-Immunodeficiency Syndrome in combination with immunosuppressive treatment	2 mg/kg/day for 4 consecutive days	Reconstitution of normal megakaryocytopoiesis and immunologic functions in Wiskott-Aldrich syndrome by marrow transplantation following myeloablation and immunosuppression with busulfan and cyclophosphamide (Kapoor et al., 1981)	Not listed in Micromedex
Cyclophosphamide	Injection/Tablet	Indication	Eczema-Thrombocytopenia-Immunodeficiency Syndrome in combination with immunosuppressive treatment	50 mg/kg/day for 4 consecutive days	Reconstitution of normal megakaryocytopoiesis and immunologic functions in Wiskott-Aldrich syndrome by marrow transplantation following myeloablation and immunosuppression with busulfan and cyclophosphamide (Kapoor et al., 1981)	Not listed in Micromedex

## 4 Conclusion

This consensus standardizes the management of off-label drug use for rare hematologic diseases, helping medical institutions develop lists of off-label drugs for rare diseases, promoting rational drug use, and addressing the diagnostic and treatment needs of patients with rare diseases. It also contributes to exploring and establishing an evaluation and management system for off-label drug use in rare diseases.
