# Supplementary material for: Expert consensus on the off-label use in China of drugs for rare hematologic diseases (2024 edition)
Source: Front Pharmacol. 2024 Nov 22;15:1477550. doi: 10.3389/fphar.2024.1477550 (PMC11621627; doi:10.3389/fphar.2024.1477550)
Supplement: Supplementary file 1 [file Table1.docx]

**Table S1**. Members of the consensus develop team

| Name | Affiliate | Title |
| --- | --- | --- |
| **Consultant** | |  |
| Zhihua Zheng | Guangdong Pharmaceutical Association | Vice Chairman and Secretary-General, Chief Pharmacist |
| Jing Sun | Nanfang Hospital, Southern Medical University | Chief Physician |
| Yilei Li | Nanfang Hospital, Southern Medical University | Chief Pharmacist |
| **Writing member** | |  |
| Boxin Zhao | Nanfang Hospital, Southern Medical University | Associate Chief Pharmacist |
| Xuan Zhou | Nanfang Hospital, Southern Medical University | Attending physician |
| **Member (in order of last name)** | |  |
| Lisheng Cai | The Second People's Hospital of Shenzhen | Chief Physician |
| Jie Chen | The First Affiliated Hospital of Sun Yat-sen University | Chief Pharmacist |
| Yilu Chen | Guangzhou Women and Children's Medical Center, Guangzhou Medical University | Chief Pharmacist |
| Shuqin Cheng | The Affiliated Panyu Central Hospital of Guangzhou Medical University | Chief Physician |
| Weiyi Feng | The First Affiliated Hospital of Xi'an Jiao Tong University | Chief Pharmacist |
| Xiaoqin Feng | Nanfang Hospital, Southern Medical University | Chief Physician |
| Liya He | Guangzhou Women and Children's Medical Center, Guangzhou Medical University | Chief Physician |
| Bo Ji | General Hospital of Southern Theatre command of PLA | Chief Pharmacist |
| Yiran Ji | The Second Hospital of Hebei Medical University | Chief Pharmacist |
| Guowei Li | Huizhou Central People’s Hospital | Chief Physician |
| Maobai Liu | Fujian Medical University Union Hospital | Chief Pharmacist |
| Taotao Liu | The First Affiliated Hospital of Guangxi Medical University | Chief Pharmacist |
| Xiaolan Mo | Guangzhou Women and Children's Medical Center, Guangzhou Medical University | Chief Pharmacist |
| Jianfen Su | The Affiliated Panyu Central Hospital of Guangzhou Medical University | Associate Chief Pharmacist |
| Junyan Wu | Sun Yat-sen Memorial Hospital, Sun Yat-sen University | Chief Pharmacist |
| Jianlong Wu | The Second People's Hospital of Shenzhen | Chief Pharmacist |
| Hui Wu | The Affiliated Hospital of Kunming Medical University | Chief Pharmacist |
| Bo Zhang | Peking Union Medical College Hospital | Chief Pharmacist |
| Hongliang Zhang | The First Affiliated Hospital of Guangxi Medical University | Chief Pharmacist |
| Yingtong Zeng | Guangdong Provincial People's Hospital Affiliated to Southern Medical University | Chief Pharmacist |
| Ping Zheng | Nanfang Hospital, Southern Medical University | Chief Pharmacist |
| Zhichang Zheng | The Affiliated Hospital of Guizhou Medical University | Chief Pharmacist |
