# Supplementary material for: Expert consensus on the off-label use in China of drugs for rare hematologic diseases (2024 edition)
Source: Front Pharmacol. 2024 Nov 22;15:1477550. doi: 10.3389/fphar.2024.1477550 (PMC11621627; doi:10.3389/fphar.2024.1477550)
Supplement: Supplementary file 2 [file Table2.docx]

# Table S2. Off-Label Drug Usage Catalog for Hemophilia Treatment

| **Generic Name** | **Dosage Form** | **Off-Label Type** | **Off-Label Content** | **Specific Usage** | **Evidence and References** | **Evidence Level** |
| --- | --- | --- | --- | --- | --- | --- |
| Human Coagulation Factor VIII | Injection | Indication  Dosage and Administr-ation | FVIII inhibitors immune tolerance therapy (ITI therapy)  Hemostatic treatment for low-titer FVIII inhibitors (congenital and acquired hemophilia): Weight (kg) × 80 × [(1 - Hct) × inhibitor titer (BU)] + 50 IU/kg | **Bonn Scheme**: Start with FVIII dose of 100 IU/kg every 12 hours, increase to 150 IU/kg every 12 hours once FVIII activity starts to recover, continue until inhibitor is resolved.  **Van Creveld Scheme**: FVIII 25-50 IU/kg every other day. Gradually reduce the dosage based on the decrease in inhibitor titer and the recovery of FVIII activity  The dosage algorithm for neutralizing inhibitors with FVIII is as follows: Weight (kg) × 80 × [(1 - Hct) × inhibitor titer (BU)]. On this basis, an additional 50 IU/kg of FVIII is required. | WFH Guidelines for the Management of Hemophilia, 3rd edition. (*Srivastava A, et al., 2020*)  Chinese Hemophilia Management Guidelines (2021) (*Hemophilia Treatment Center Collaboration Network of China, 2021*)  Chinese Guidelines for Hemophilia Treatment (2020). (*Thrombosis and Hemostasis Group, et al, 2020*)  Chinese Guidelines for Diagnosis and Treatment of Factor VIII/IX Inhibitors (2018) (*Thrombosis and Hemostasis Group, et al, 2018*)  WFH Guidelines for the Management of Hemophilia, 3rd edition. (*Srivastava A, et al., 2020*)  Chinese Hemophilia Management Guidelines (2021) (*Hemophilia Treatment Center Collaboration Network of China, 2021*)  Chinese Guidelines for Hemophilia Treatment (2020). (*Thrombosis and Hemostasis Group, et al, 2020*) | Not listed in Micromedex  Not listed in Micromedex |
| Recombinant Human Factor VIII | Injection | Indication  Dosage and Administration | FVIII inhibitors immune tolerance therapy (ITI therapy)  Hemostatic treatment for low-titer FVIII inhibitors (congenital and acquired hemophilia): Weight (kg) × 80 × [(1 - Hct) × inhibitor titer (BU)] + 50 IU/kg | **Bonn protocol**: Start with FVIII 100 IU/kg every 12 hours, once FVIII activity begins to recover, increase to 150 IU/kg every 12 hours until the inhibitor is eradicated;  **Van** **Creveld scheme**: FVIII 25-50 IU/kg every other day, adjust dosage based on inhibitor titer decrease and FVIII activity recovery.  The dosage algorithm for neutralizing inhibitors with FVIII is as follows: Weight (kg) × 80 × [(1 - Hct) × inhibitor titer (BU)]. On this basis, an additional 50 IU/kg of FVIII is required. | WFH Guidelines for the Management of Hemophilia 3rd edition, (*Srivastava A, et al., 2020*)  Chinese Guidelines for Hemophilia Management (2021) (*Hemophilia Treatment Center Collaboration Network of China, 2021*)  Chinese Guidelines for Hemophilia Treatment (2020) (*Thrombosis and Hemostasis Group, et al, 2020*)  Chinese Guidelines for Diagnosis and Treatment of Factor VIII/IX Inhibitors (2018) (*Thrombosis and Hemostasis Group, et al, 2018*)  WFH Guidelines for the Management of Hemophilia, 3rd edition. (*Srivastava A, et al., 2020*)  Chinese Hemophilia Management Guidelines (2021) (*Hemophilia Treatment Center Collaboration Network of China, 2021*)  Chinese Guidelines for Hemophilia Treatment (2020). (*Thrombosis and Hemostasis Group, et al, 2020*)  Chinese Guidelines for Diagnosis and Treatment of Factor VIII/IX Inhibitors (2018) (*Thrombosis and Hemostasis Group, et al, 2018*) | Not listed in Micromedex  Not listed in Micromedex |
| Human Prothrombin Complex | Injection | Indication and Dosage | Treatment of bleeding in acquired FVIII inhibitor states via bypass pathway: 50-75 IU/kg | For general joint bleeds, dose can be 50-75 IU/kg per occasion, increase to 100 IU/kg for severe or life-threatening bleeds, but not exceeding 150 IU/kg in 24 hours. | Chinese Guidelines for Hemophilia Management (2021) (*Hemophilia Treatment Center Collaboration Network of China, 2021*)  Chinese Guidelines for Diagnosis and Treatment of Acquired Hemophilia A (2021) (*Thrombosis and Hemostasis Group, et al, 2021*) | Not listed in Micromedex |
|  |  |  |  |  |  |  |
| Emicizumab | Injection | Indication | Routine prophylactic treatment for A-type hemophilia patients without FVIII inhibitors, adults and children | First 4 Weeks: 3 mg/kg weekly; After Week 4: 1.5 mg/kg weekly, or 3 mg/kg every two weeks, or 6 mg/kg every four weeks | FDA approved (*Product Information: HEMLIBRA(R) subcutaneous injection, emicizumab-kxwh subcutaneous injection, 2021*) | Effectiveness Class IIa, Recommendation Class IIa  Evidence Category B |
|  |  |  |  |  |  |  |
| Tranexamic Acid | Injection/Tablet  Tablet | Indication  Administr-ation | Prevention of bleeding in hemophilia patients during tooth extraction  Rinse | Injection: Pre-operative 10 mg/kg IV, single dose, with replacement therapy, immediately followed by tooth extraction. Post-operative: 10 mg/kg IV, three times daily, treatment duration 2-8 days.  Take orally, rinse mouth, or crush the tablet, dissolve in water and use for local mucosal bleeding. | FDA approved (*Product Information: CYKLOKAPRON(R) intravenous injection, tranexamic acid intravenous injection, 2020*)  WFH Guidelines for the Management of Hemophilia, 3rd edition(*Srivastava A, et al., 2020*).  Chinese Hemophilia Management Guidelines (2021) (*Hemophilia Treatment Center Collaboration Network of China, 2021*).  WFH Guidelines for the Management of Hemophilia, 3rd edition (*Srivastava A, et al., 2020*).  Chinese Hemophilia Management Guidelines (2021) (*Hemophilia Treatment Center Collaboration Network of China, 2021*). | Effectiveness Class IIa, Recommendation Class IIa  Evidence Category B  Not listed in Micromedex |
| Prednisone | Tablet | Indication | 1. First-line immunosuppressive treatment for acquired hemophilia;  2. Combined immunosuppressive therapy in immune tolerance treatment for congenital hemophilia with inhibitors | 1. Prednisone 1 mg/kg/day orally or equivalent dose of other corticosteroids orally or IV, typically not exceeding 6 weeks, gradually tapered to discontinuation.  2. Combined with Bonn Scheme, including oral prednisone 50-150 mg/day. | 1. WFH Guidelines for the Management of Hemophilia, 3rd edition (*Srivastava A, et al., 2020*).  2. Chinese Guidelines for Diagnosis and Treatment of Acquired Hemophilia A (2021) (*Thrombosis and Hemostasis Group, et al, 2021*).  3. Chinese Hemophilia Management Guidelines (2021) (*Hemophilia Treatment Center Collaboration Network of China, 2021*). | Not listed in Micromedex |
|  |  |  |  |  |  |  |
| Rituximab | Injection | Indication | 1. Combined first-line immunosuppressive therapy for acquired hemophilia;  2. Second-line therapy for congenital hemophilia with inhibitors in immune tolerance induction therapy | 1. Combined with corticosteroids; 375 mg/m² weekly, IV, up to 4 times or 100 mg weekly x 4 weeks.  2. Second-line treatment in immune tolerance induction therapy for congenital hemophilia with FVIII inhibitors: Recommended dose of rituximab is 375 mg/m² weekly, for a maximum of 4 cycles | 1. WFH Guidelines for the Management of Hemophilia, 3rd edition (*Srivastava A, et al., 2020*).  2. Chinese Guidelines for Diagnosis and Treatment of Acquired Hemophilia A (2021) (*Thrombosis and Hemostasis Group, et al, 2021*)  3. Reduced-intensity, risk factor-stratified immunosuppression for acquired hemophilia A: single-center observational study(*Dobbelstein C, et al., 2020*)  4. Chinese Guidelines for the Treatment of Hemophilia (2020 Edition) (*Thrombosis and Hemostasis Group, et al, 2020*)  5. Chinese Guidelines for the Diagnosis and Treatment of Factor VIII/IX Inhibitors (2018 Edition) (*Thrombosis and Hemostasis Group, et al, 2018*) | Not listed in Micromedex |
| Cyclophosphamide | Injection/Tablet | Indication | 1. Combined first-line immunosuppressive therapy for acquired hemophilia;  2. Second-line therapy for congenital hemophilia with inhibitors in immune tolerance induction therapy | 1. Combined with corticosteroids; Cyclophosphamide 1.5-2 mg/kg/day, IV or orally, typically not exceeding 6 weeks.  2. In combination with Bonn Scheme, cyclophosphamide 12-15 mg/(kg·d) IV for 2 days followed by 2-3 mg/(kg·d) orally for 8-10 days | 1. WFH Guidelines for the Management of Hemophilia, 3rd edition (*Srivastava A, et al., 2020*).  2. Chinese Guidelines for Diagnosis and Treatment of Acquired Hemophilia A (2021) (*Thrombosis and Hemostasis Group, et al, 2021*).  3. Reduced-intensity, risk factor-stratified immunosuppression for acquired hemophilia A: single-center observational study (*Dobbelstein C, et al., 2020*)  4. Chinese Guidelines for the Treatment of Hemophilia (2020 Edition) (*Thrombosis and Hemostasis Group, et al, 2020*)  5. Chinese Guidelines for the Diagnosis and Treatment of Factor VIII/IX Inhibitors (2018 Edition) (*Thrombosis and Hemostasis Group, et al, 2018*) | Effectiveness Class IIb, Recommendation Class IIB.  Evidence Category C |
| Human Immunoglobulin | Injection | Indication | Second-line therapy in immune tolerance induction treatment for congenital hemophilia with inhibitors | In combination with Bonn Scheme, including IV administration of human immunoglobulin 0.4 g/(kg·d) for 5 days | 1. WFH Guidelines for the Management of Hemophilia, 3rd edition (*Srivastava A, et al., 2020*).  2. Chinese Guidelines for the Treatment of Hemophilia (2020 Edition) (*Thrombosis and Hemostasis Group, et al, 2020*)  3. Chinese Guidelines for Diagnosis and Treatment of Acquired Hemophilia A (2021) (*Thrombosis and Hemostasis Group, et al, 2021*).  3. Chinese Guidelines for the Diagnosis and Treatment of Factor VIII/IX Inhibitors (2018 Edition) (*Thrombosis and Hemostasis Group, et al, 2018*) | Not listed in Micromedex |
|  |  |  |  |  |  |  |
| Azathioprine | Tablet | Indication | Immunosuppressive therapy for acquired hemophilia | Used when first line and second-line therapies are ineffective. | 1. WFH Guidelines for the Management of Hemophilia, 3rd edition(*Srivastava A, et al., 2020*).  2. Chinese Guidelines for the Treatment of Hemophilia (2020 Edition) (*Thrombosis and Hemostasis Group, et al, 2020*)  3. Chinese Guidelines for Diagnosis and Treatment of Acquired Hemophilia A (2021) (*Thrombosis and Hemostasis Group, et al, 2021*).  3. Chinese Guidelines for the Diagnosis and Treatment of Factor VIII/IX Inhibitors (2018 Edition) (*Thrombosis and Hemostasis Group, et al, 2018*) | Not listed in Mimromedex |
|  |  |  |  |  |  |  |
| Vincristine | Injection | Indication | Immunosuppressive therapy for acquired hemophilia | Used when first line and second-line therapies are ineffective. | 1. WFH Guidelines for the Management of Hemophilia, 3rd edition (*Srivastava A, et al., 2020*).  2. Chinese Guidelines for the Treatment of Hemophilia (2020 Edition) (*Thrombosis and Hemostasis Group, et al, 2020*)  3. Chinese Guidelines for Diagnosis and Treatment of Acquired Hemophilia A (2021) (*Thrombosis and Hemostasis Group, et al, 2021*).  3. Chinese Guidelines for the Diagnosis and Treatment of Factor VIII/IX Inhibitors (2018 Edition) (*Thrombosis and Hemostasis Group, et al, 2018*) | Not listed in Micromedex |
|  |  |  |  |  |  |  |
| Mycophenolate Mofetil | Injection/Tablet/Capsule | Indication | Immunosuppressive therapy for acquired hemophilia | Used when first line and second-line therapies are ineffective. | 1. WFH Guidelines for the Management of Hemophilia, 3rd edition (*Srivastava A, et al., 2020*).  2. Chinese Guidelines for the Treatment of Hemophilia (2020 Edition) (*Thrombosis and Hemostasis Group, et al, 2020*)  3. Chinese Guidelines for Diagnosis and Treatment of Acquired Hemophilia A (2021) (*Thrombosis and Hemostasis Group, et al, 2021*).  3. Chinese Guidelines for the Diagnosis and Treatment of Factor VIII/IX Inhibitors (2018 Edition) (*Thrombosis and Hemostasis Group, et al, 2018*) | Not listed in Micromedex |
|  |  |  |  |  |  |  |
| Cyclosporine A | Injection/Soft Capsule/Oral Solution | Indication | Immunosuppressive therapy for acquired hemophilia | Used when first line and second-line therapies are ineffective. | 1. WFH Guidelines for the Management of Hemophilia, 3rd edition (*Srivastava A, et al., 2020*).  2. Chinese Guidelines for the Treatment of Hemophilia (2020 Edition) (*Thrombosis and Hemostasis Group, et al, 2020*)  3. Chinese Guidelines for Diagnosis and Treatment of Acquired Hemophilia A (2021) (*Thrombosis and Hemostasis Group, et al, 2021*).  3. Chinese Guidelines for the Diagnosis and Treatment of Factor VIII/IX Inhibitors (2018 Edition) (*Thrombosis and Hemostasis Group, et al, 2018*) | Not listed in Micromedex |
|  |  |  |  |  |  |  |
| Chromic Phosphate [32P] Colloidal | Injection | Indication | Radionuclide synovectomy in hemophilia patients | Adult dose for knee, hip joints: 1.0-2.0 mCi; for ankle, elbow joints: 0.5-1.0 mCi. For children (or weight less than 30 kg): dose halved. | 1. WFH Guidelines for the Management of Hemophilia, 3rd edition (*Srivastava A, et al., 2020*).  2. Chinese Hemophilia Management Guidelines (2021) (*Hemophilia Treatment Center Collaboration Network of China, 2021*). | Not listed in Micromedex |
|  |  |  |  |  |  |  |

**References**

1. Srivastava A, Santagostino E, Dougall A, Kitchen S, Sutherland M, Pipe SW, et al. (2020). WFH Guidelines for the Management of Hemophilia, 3rd edition. Haemophilia, 26(S6):1-158
2. Hemophilia Treatment Center Collaboration Network of China (2021). Chinese Guidelines for the management of Hemophilia (2021 Edition). Peking Union Medical College Press, Nov. 2021, 1st Edition
3. Thrombosis and Hemostasis Group, Chinese Society of Hematology, Chinese Medical Association; Hemophilia Treatment Center Collaborative Network of China. (2020). Chinese guidelines on the treatment of hemophilia (version 2020). Chin J Hematol, 41(4):265-271.
4. Thrombosis and Hemostasis Group, Chinese Society of Hematology, Chinese Medical Association, Hemophilia Treatment Center Collaborative Network of China. (2018). Chinese guidelines on the diagnosis and treatment of coagulation factor Ⅷ/Ⅸ inhibitors (version 2018), Chinese Journal of Hematology, 39(10):793-799
5. Thrombosis and Hemostasis Group, Chinese Society of Hematology, Chinese Medical Association; Hemophilia Treatment Center Collaborative Network of China.(2021). Chinese guidelines on the diagnosis and treatment of acquired hemophilia A (2021). Chin J Hematol, 42(10): 793-799.
6. Dobbelstein C, Moschovakis GL, Tiede A. (2020). Reduced-intensity, risk factor-stratified immunosuppression for acquired hemophilia A: single-center observational study. Ann Hematol, 99(9): 2105-2112.
