# Supplementary material for: Expert consensus on the off-label use in China of drugs for rare hematologic diseases (2024 edition)
Source: Front Pharmacol. 2024 Nov 22;15:1477550. doi: 10.3389/fphar.2024.1477550 (PMC11621627; doi:10.3389/fphar.2024.1477550)
Supplement: Supplementary file 3 [file DataSheet1.docx]

| **Effective Level** | | |
| --- | --- | --- |
| Ⅰ | Effective | □ |
| Ⅱa | Evidence Favors Efficacy | □ |
| Ⅱb | Evidence is Inconclusive | □ |
| Ⅲ | Ineffective | □ |

| **Recommendation Level** | | |
| --- | --- | --- |
| ClassⅠ | Recommended | □ |
| Class Ⅱa | Recommended in Most Cases | □ |
| Class Ⅱb | Recommended in Some Cases | □ |
| Class Ⅲ | Not Recommended | □ |
| Class Indeterminate | Indeterminate | □ |

**Appendix 1. Off-Label Drug Use Evidence-Based Evaluation Form**

| **Off-Label Drug Use Content：**  Drug Name： Drug Dosage Form：  Off-Label Use Content： | | | | | | | | |
| --- | --- | --- | --- | --- | --- | --- | --- | --- |
| **Off-Label Use Category**  □Beyond Indication □Beyond Dosage □Beyond Administration Route □Beyond Patient Population □Other | | | | | | | | |
| **Summary of Evidence Review**  □Original National Drug Package Insert Approved □Micromedex Included □Medical Literature (Note: Not approved in original drug package inserts and not included in Micromedex, only mentioned in other literature) | | | | | | | | |
| **Original National Drug Package Insert Evidence Rivew** □Yes □No | | | | | | | | |
|  | Generic Name | Dosage Form | Specification | Manufacturer | Approval country | Approval Date | Approval Content |  |
|  |  |  |  |  |  |  |  |  |
|  |  |  |  |  |  |  |  |  |
| **Micromedex Database Evidence Review**  □Yes □No  Micromedex Database Evidence Review Conclusion：   \| **Evidence Level** \| \| \| \| --- \| --- \| --- \| \| Category A \| Evidence based on the following: meta-analyses of randomized controlled trials; multiple, well-designed, large-scale randomized clinical trials \| □ \| \| Category B \| Evidence based on the following: meta-analyses of conflicting randomized controlled trials; small-scale or methodologically flawed randomized controlled trials; non-randomized studies. \| □ \| \| Category C \| Evidence based on the following: expert opinion or consensus; case reports or series \| □ \| | | | | | | | | |

6

**Medical Literature Evidence Evaluation Form**

**Type of Medical Literature Evidence Review**

□Strong Recommendation Re

□Weak Recommendation

| **Type of Medical Literature** | **Number of Included in Evidence Review** | **Efficacy Evaluation Conclusion** | **Safety Evaluation Conclusion** |
| --- | --- | --- | --- |
| Clinical Guidelines/Expert Consensus |  |  |  |
| Systematic Review |  |  |  |
| Randomized Controlled Trials |  |  |  |
| Non-Randomized Controlled Trials |  |  |  |
| Cohort Studies |  |  |  |
| Case-Control Studies |  |  |  |
| Case Reports |  |  |  |
| Medical Books |  |  |  |
| Other |  |  |  |

Summary of Evidence-Based Review Conclusions for Medical Literature

Summary of Efficacy Evaluation Conclusion

Summary of Safety Evaluation Conclusion

**Comprehensive Recommendation for Off-Label Drug Use Evidence-Based Evaluation**

| **Evaluator** |  | **Reviewer** |  |
| --- | --- | --- | --- |
| **Evaluation Date** |  | **Review Date** |  |

7
